# *APOE* ε4 influences the widespread TDP-43 pathological subtype in sporadic amyotrophic lateral sclerosis

**DOI:** 10.1007/s00401-026-03029-y

**Published:** 2026-05-15

**Authors:** Yuya Hatano, Asa Nakahara, Mari Tada, Akiyoshi Kakita, Osamu Onodera, Tomohiko Ishihara

**Affiliations:** 1 Department of Neurology, Brain Research Institute, Niigata University, Niigata, Japan; 2 Department of Neurology, Uonuma Institute of Community Medicine, Niigata University Medical and Dental Hospital, Minamiuonuma, Japan; 3 Department of Pathology, Brain Research Institute, Niigata University, Niigata, Japan; 4 Center for Human Brain Resource Initiative (ChBRI), Niigata University, Niigata, Japan; 5 Advanced Treatment of Neurological Diseases Branch, Endowed Research Branch, Brain Research Institute, Niigata University, 1-757 Asahimachi-Dori, Chuo-Ku, Niigata, 951-8585 Japan

**Keywords:** APOE, Amyotrophic lateral sclerosis, TDP-43 pathology, Amyloid-β, Tau, Structural equation modeling

## Abstract

Amyotrophic lateral sclerosis (ALS) is a fatal neurodegenerative disorder, most sporadic cases exhibiting TAR DNA-binding protein 43 (TDP-43) pathology. The anatomical distribution of TDP-43 pathology varies among patients; however, factors contributing to this heterogeneity remain unclear. Apolipoprotein E (*APOE*) ε4 is known to influence the spread of pathological protein in several neurodegenerative diseases, raising the possibility that it also modulates the pathological distribution of TDP-43 inclusions in ALS. We investigated this hypothesis in a cohort of 145 autopsy-confirmed sporadic ALS cases. ALS-associated TDP-43 pathology was classified into two subtypes: type 1 – largely restricted to motor regions – and type 2 – characterized by widespread cortical involvement. *APOE* genotypes and rare variants in known ALS-associated genes were determined by exome sequencing. Amyloid-β and tau pathologies were assessed neuropathologically using established staging systems. Structural equation modeling (SEM) was applied to disentangle direct and indirect relationships among *APOE* ε4, temporal clinical parameters, Alzheimer’s disease-related pathologies, and ALS TDP-43 subtype. Furthermore, we also performed an unbiased evaluation using random forest model. *APOE* ε4 carriers showed a significantly higher proportion of type 2 pathology than non-carriers. Bayesian SEM demonstrated that APOE ε4 was directly associated with the type 2, widespread TDP-43 subtype, independent of amyloid-β and tau pathology, while also reproducing the canonical cascade linking *APOE* ε4 to amyloid-β and tau. Rare variants in ALS-associated genes showed no clear effect on TDP-43 subtype. These findings indicate that *APOE* ε4 modifies the anatomical distribution of TDP-43 pathology in sporadic ALS through mechanisms independent of classical Alzheimer’s disease pathology. Incorporation of *APOE* genotype into ALS stratification may be informative for biologically grounded subtype-specific therapeutic approaches.

**Supplementary Information:**

The online version contains supplementary material available at 10.1007/s00401-026-03029-y.

## Introduction

Amyotrophic lateral sclerosis (ALS) is a neurodegenerative disease characterized by progressive degeneration of upper and lower motor neurons, leading to death typically within 2–4 years after symptom onset [1]. Approximately 5–10% of ALS cases are familial (FALS), while the remainder are sporadic (SALS). The pathological hallmark in the vast majority of sporadic ALS cases is the presence of TAR DNA-binding protein 43 (TDP-43)-positive inclusions in neurons and/or glial cells [2, 3].

We previously described TDP-43 pathology in ALS on the basis of its anatomical distribution, using two major patterns: type 1, in which TDP-43 inclusions are largely confined to the motor system, and type 2, in which inclusions extend beyond the motor system to involve the frontotemporal cortex, hippocampal formation, neostriatum, and substantia nigra [4]. Cases with type 2 pathology frequently present with dementia [4]. At the same time, a neuropathological staging model has proposed that phosphorylated TDP-43 pathology in ALS may propagate sequentially along axonal pathways, raising the possibility that cases with temporal cortex and hippocampal involvement could represent a more advanced stage of disease rather than a distinct pathological subtype [5]. Notably, the extent and distribution of TDP-43 inclusions cannot be solely explained by disease duration [4], indicating that additional factors may influence both the distribution pattern and progression of TDP-43 pathology in ALS. However, the factors responsible for the observed distributional heterogeneity of TDP-43 inclusions remain largely unidentified.

As is the case with ALS, many other neurodegenerative diseases are characterized by the accumulation of disease-specific proteins within neural tissues. The distribution and progression of such pathological protein accumulation are influenced by apolipoprotein E (*APOE*) allelic polymorphisms. In Alzheimer’s disease (AD), the *APOE* ε4 allele is not only a major genetic risk factor for disease onset [6, 7], but is also associated with the pathological spread of amyloid-β and tau protein [8]. Furthermore, studies across multiple neurodegenerative disease pathologies have demonstrated that *APOE* ε4 promotes the dissemination of Lewy body pathology [8].

Recent evidence has increasingly indicated a correlation between *APOE* ε4 and TDP-43 pathology. In pathological studies of AD, *APOE* ε4 has been identified as a risk factor for the formation of TDP-43 inclusions [9]. Structural equation modeling (SEM) analyses have shown that *APOE* ε4 contributes to TDP-43 inclusion formation either directly or indirectly through amyloid-β and tau pathology [9].

Large-scale proteomics analyses have further broadened the biological interpretation of *APOE* ε4 beyond AD [10]. Using CSF, plasma, and brain proteomes from over 11,000 individuals, a conserved *APOE* ε4-associated protein signature enriched for pro-inflammatory immune pathways was identified across multiple neurodegenerative diseases and non-impaired controls. Importantly, this ε4-associated phenotype appeared to be largely independent of clinical diagnosis and classical neuropathologies, suggesting a disease-independent biological vulnerability conferred by *APOE* ε4.

Taken together, these findings raise the possibility that *APOE* ε4 modifies the anatomical distribution of ALS-associated TDP-43 pathology. To test this, we applied SEM and random forest analysis to disentangle the relationship between *APOE* ε4 and ALS TDP-43 pathological subtype while accounting for amyloid-β and tau pathology as well as age at death.

## Methods

### Participants

The study included individuals with sporadic ALS who were autopsied between 1978 and 2023 at the Department of Pathology, Brain Research Institute, Niigata University, Japan, and were diagnosed neuropathologically as having ALS. Eligibility criteria for cases were the presence of TDP-43-positive inclusions in the central nervous system and provision of consent for DNA analysis. Cases in which the onset of dementia preceded the onset of motor symptoms by more than one year were excluded. Among the cases included in the present study, 75 had already been analyzed and included in a previous report [11].

### Genetic analysis

Exome sequencing was performed to identify variants in known ALS causative genes, and APOE genotype was also determined from the exome sequencing data. DNA samples were extracted from autopsied central nervous system tissues (occipital cortex, motor cortex, and cerebellum) using a DNA extraction kit (QIAamp DNA Mini Kit; Bio-Rad Laboratories, Hercules, CA, USA). Exome sequencing was outsourced to Takara Bio (Shiga, Japan) or Macrogen (Seoul, South Korea) and performed using the Illumina NovaSeq 6000 platform. Analysis of GGGGCC repeat expansions in intron 1 of *C9orf72* was performed in all cases using previously reported methods [12].

Annotated exome sequencing data were used to detect variants in known ALS-associated genes, including *TARDBP*, *OPTN*, *FUS*, *SOD1*, *TBK1*, *SQSTM1*, *MATR3*, *TUBA4A*, *NEK1*, *HNRNPA2B1*, *VCP*, *ELP3*, *SETX*, *HNRNPA1*, *CCNF*, *VAPB*, *C21orf2*, *CHCHD10*, *NEFH*, *ANG*, *DCTN1*, *CHMP2B*, *UBQLN2*, *FIG4*, *PFN1*, *EWSR1*, *TAF15*, *ANXA11*, *DAO*, *ERBB4*, *TIA1*, *GLE1*, *PRPH*, *ALS2*, *SPG11*, *SIGMAR1*, *KIF5A*, and *DNAJC7*. Given that the allele frequency of the well-established pathogenic variant *SOD1* p.D91A is 0.001 across all populations in the gnomAD database [13], non-synonymous variants with an allele frequency of < 0.001 were defined as rare variants (RV) and included in the analysis. All detected rare variants were validated by Sanger sequencing regardless of sequencing depth. Allele frequency data were obtained from the Human Genetic Variation Database (HGVD; http://www.hgvd.genome.med.kyoto-u.ac.jp/) and the Exome Aggregation Consortium databases (ExAC All / East Asian populations; https://gnomad.broadinstitute.org/). For autosomal recessive ALS-associated genes (*ALS2*, *SPG11*, and *SIGMAR1*), only homozygous variants were considered.

*APOE* genotypes were determined using annotated exome sequencing data, with alleles defined by amino acid residues at positions 112 and 158: ε2 (Cys112/Cys158), ε3 (Cys112/Arg158), and ε4 (Arg112/Arg158) [14]. *APOE* genotypes were assigned based on the combination of these alleles.

### Neuropathological analysis

**Fig. 1:**
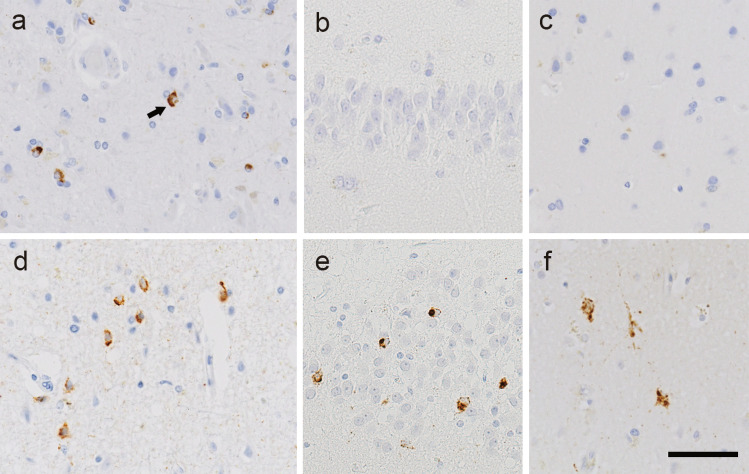
Representative TDP-43 pathology of ALS type 1 and type 2. a—c. Phosphorylated TDP-43 (pTDP-43) immunohistochemistry in a patient with ALS type 1. The motor cortex shows a few neuronal cytoplasmic inclusions (NCIs) and scattered glial cytoplasmic inclusions (**a**). No pTDP-43-immunoreactive inclusions are evident in the hippocampal granule cells (**b**) or temporal cortex (**c**). Arrow, NCI. d—f. pTDP-43 immunohistochemistry in a patient with ALS type 2. Abundant NCIs are evident in the motor cortex (**d**), hippocampal granule cells (**e**), and temporal cortex (**f**). Bar = 50 μm

Immunohistochemical analyses were performed on 4-μm-thick sections prepared from formalin-fixed, paraffin-embedded blocks obtained from multiple regions of the central nervous system, including the frontal cortex, motor cortex, temporal cortex, hippocampus medulla oblongata, and spinal cord. ALS-associated TDP-43 pathology was evaluated using immunohistochemistry with an antibody against phosphorylated TDP-43 (pTDP-43; pS409/410). We previously demonstrated that the majority of patients with sporadic ALS can be classified into two pathological types (type 1 and type 2, as mentioned above) based on the presence or absence of TDP-43 neuronal cytoplasmic inclusions (NCIs) in the hippocampal dentate gyrus [4]. Accordingly, all cases in the present study were classified using this criterion. Representative pTDP-43 immunohistochemistry images in type 1 and type 2 patients are presented in Fig. 1. In patients with both types, pTDP-43 NCIs and GCIs were observed in the motor cortex (Fig. 1a, d). By contrast, in type 1 patients, they were absent from the hippocampal dentate granule cells (Fig. 1b) and exceedingly rare in the temporal cortex (Fig. 1c). In type 2 patients, however, NCIs and GCIs were present in varying numbers in these regions (Fig. 1e, f). AD-related pathological changes were staged according to the criteria proposed by Thal et al. for senile plaques and by Braak et al. for neurofibrillary tangles (NFT) [15–17]. Immunohistochemistry for amyloid-β and phosphorylated tau was performed on temporal lobe sections including the hippocampus, with additional staining of the frontal cortex, motor cortex, and occipital cortex as needed. The primary antibodies used in this study are listed in Supplementary Table 1.

### Statistical analysis

The primary analytical approaches used in this study were Bayesian SEM and random forest analysis. SEM was employed to simultaneously evaluate the complex relationships among genetic factors, AD-related pathologies, and temporal clinical parameters, and to estimate their direct and indirect effects on the distribution of TDP-43 pathology. In the present study, several endogenous variables were ordinal in nature, and the sample size was relatively small; therefore, Bayesian SEM was adopted, as it is known to provide more stable parameter estimates than conventional maximum likelihood estimation under these conditions. In addition, random forest analysis was used as a complementary approach. This nonparametric machine learning method does not require a priori assumptions regarding causal pathways and is capable of capturing nonlinear relationships and interactions among variables.

Determinants of TDP-43 pathological subtype (type 1 vs. type 2) were evaluated using both SEM and random forest models. The overall analytical workflow is summarized as follows. Two analytical frameworks were constructed by varying the definitions of temporal clinical parameters: model (a) and model (b). In both random forest models, the explanatory variables included Thal Aβ phase, Braak NFT stage, APOE4 status, and the presence of RV. Temporal clinical parameters were defined as follows: in model (a), age at death and entire disease course were included, whereas in model (b), age at onset and survival time were used. “Entire course” was defined as the interval from symptom onset to death, including the period after initiation of mechanical ventilation. “Survival time” was defined as the interval from symptom onset to death or initiation of mechanical ventilation. The hypothesized pathways among variables in the SEM analyses for models (a) and (b) are illustrated in Fig. 3a and b, respectively. In model (a), a final SEM model was constructed by retaining only the paths whose 95% credible intervals did not include zero. In addition, determinants of age at death were evaluated using both SEM and random forest models. In this random forest analysis, the explanatory variables included APOE4 status, Thal Aβ phase, Braak NFT stage, TDP-43 pathology type (type 2), and RV. The corresponding SEM pathways are shown in Fig. 3.

Categorical variables are summarized as counts and percentages, and continuous variables as medians and ranges. Comparisons between the TDP-43 pathology type 1 and type 2 groups were performed using Fisher’s exact test for categorical variables, the Wilcoxon rank-sum test for continuous variables, and the log-rank test for survival time.

For SEM, variables were defined as follows. *APOE* genotypes ε2/ε4 and ε3/ε4 were coded as APOE4 = 1, ε4/ε4 as APOE4 = 2, and all other genotypes as APOE4 = 0. The presence of at least one RV in known ALS-associated genes was coded as RV = 1, and the absence of such variants as RV = 0. The Thal Aβ phase, Braak NFT stage, and TDP-43 pathological types were classified according to their respective classification systems.

Bayesian SEM was used to accommodate ordinal endogenous variables and a relatively small sample size [18]. Bayesian estimation was performed using the *blavaan* package in R. All endogenous variables, except for age at death, were treated as ordinals in the *blavaan* analysis. Standardized path coefficients were estimated, and 95% credible intervals (posterior intervals, indicating a 95% probability that the true value lies within the interval) were calculated. Effects whose 95% credible intervals did not include zero were considered statistically significant. Model fit was evaluated using the posterior predictive p-value (PPP), and PPP values between 0.1 and 0.9 were considered to indicate an acceptable model fit.

To assess the robustness of the results, SEM (a) model was also estimated using the unweighted least squares parameter with mean- and variance-adjusted chi-squared test statistic (ULSMV). Standardized coefficients and p values were calculated. ULSMV estimation was performed using the *lavaan* package in R. For ULSMV analyses, missing data were imputed using the *mice* package, with the number of imputations set to *m* = 20. For missing data imputation using the *mice* package, the predictive mean matching (pmm) method was applied to continuous variables (age at death and entire course), whereas the proportional odds logistic regression (polr) method was used for ordinal variables.

Random forest models were evaluated using five-fold cross-validation. To prevent data leakage, missing data imputation was performed after splitting the data into training and validation folds. Specifically, the imputation model was fitted using only the training data with the *mice* package and then applied to both the training and validation folds. The random forest model was trained on the training fold and evaluated on the validation fold. Model performance was assessed using root mean squared error (RMSE), mean absolute error (MAE), and R^2^ for the analysis of determinants of age at death, and area under the receiver operating characteristic curve (AUC) for the classification of TDP-43 pathological type. The average performance across the five folds was used as the final evaluation metric. For missing data imputation, pmm was applied to continuous variables (age at death, entire course, age at onset, and survival time), whereas polr was used for ordinal variables. The number of imputations was set to m = 20. To visualize the effects of individual variables, partial dependence plots (PDPs) were generated by averaging the effects of other variables. PDPs were constructed using the combined training and validation data, with predictions averaged across the 20 imputed datasets and further averaged over the five folds. Variable importance was assessed using the percent increase in mean squared error (%IncMSE) for the regression task (age at death) and Mean Decrease in Accuracy for the classification task (TDP-43 pathology type).

All statistical analyses were conducted using R software version 4.4.0.

## Results

### Dataset details

**Fig. 2:**
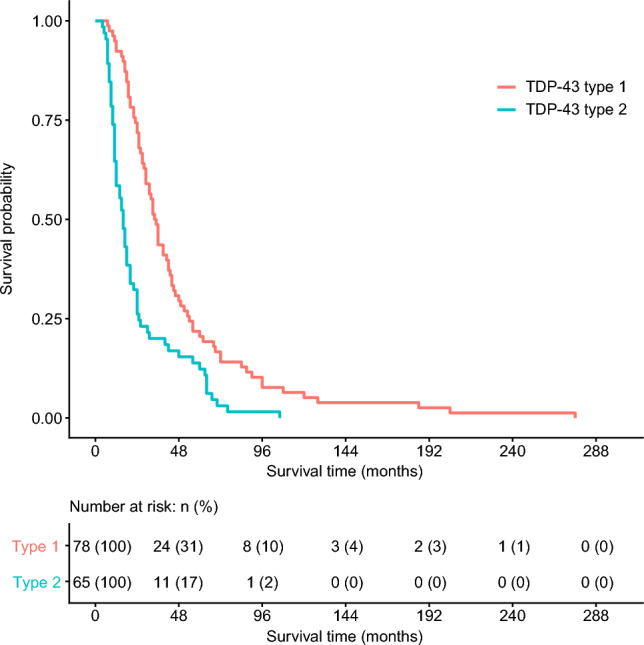
Kaplan–Meier survival curves for overall survival stratified by APOE genotype. Numbers at risk are shown below the x-axis

A total of 145 neuropathologically confirmed cases of sporadic ALS were included in this study. Clinical characteristics, APOE genotypes, and neuropathological features are summarized in Table 1. The median age at onset was 64.6 years (range, 34—89 years), the median age at death was 70 years (range, 45—94 years), the median entire disease course was 36 months (range, 7—437 months), and the median survival time was 25 months (range, 4—276 months). The cases with dementia accounted for 28 of 88 (25.9%), and the cases with bulbar onset accounted for 42 of 143 (29.4%). According to the ALS TDP-43 pathology classification, 80 cases (55.2%) were classified as type 1 and 65 cases (44.8%) as type 2. Compared with type 1 cases, type 2 cases had a significantly older age at onset (Wilcoxon rank sum test, p = 0.00031), and age at death (Wilcoxon rank sum test, p = 0.0016). Type 2 cases exhibited a significantly shorter survival time from onset to death or the initiation of ventilatory support compared with type 1 cases, indicating a poorer prognosis (excluding two cases with incomplete clinical data; log-rank test, p = 0.000004; Table 1, Fig. 2). In addition, the frequency of dementia was significantly higher in type 2 than in type 1 (Fisher’s exact test, p = 0.000010; Table 1). In contrast, there was no significant difference in the frequency of bulbar onset between types 1 and 2 (Fisher’s exact test, p = 0.20; Table 1).

**Table 1:** Characteristics of the study participants

	No. (%) or median (range)				
	All (N = 145)	Type 1 (N = 80)	Type 2 (N = 65)	odds ratio [95% CI]	p value
Female	56 (38.6)	30 (37.5)	26 (40.0)	1.11 [0.54—2.29]	0.86
Age at onset, y	64.6 (34–89)	61.2 (37–86.2)	69 (34–89)	–	0.00031
Age at death, y	70 (45–94)	67.5 (45–87.83)	74 (54–94)	–	0.0016
Survival time, m	25 (4–276)	34.5 (7–276)	16 (4–106)	–	0.000004
Entire course, m	36 (7–437)	44.5 (7—437)	24 (7–228)	–	0.0048
Dementia	28 of 88 (25.9)	5 of 46 (10.9)	23 of 42 (54.8)	9.64 [2.99–37.57]	1.04E-05
Bulbar onset	42 of 143 (29.4)	19 of 78 (24.4)	23 of 65 (35.4)	1.69 [0.77–3.75]	0.20
APOE					
ε2/ε2	1 (0.7)	1 (1.3)	0 (0)	–	0.074
ε2/ε3	13 (9.0)	7 (8.8)	6 (9.2)		
ε2/ε4	2 (1.4)	0 (0)	2 (3.1)		
ε3/ε3	102 (70.3)	62 (77.5)	40 (61.5)		
ε3/ε4	26 (17.9)	10 (12.5)	16 (24.6)		
ε4/ε4	1 (0.7)	0 (0)	1 (1.5)		
APOE ε2 carrier	16 (11.0)	8 (10.0)	8 (12.3)	1.26 [0.39–4.12]	0.79
APOE ε4 carrier	29 (20.0)	10 (12.5)	19 (29.2)	2.87 [1.14–7.58]	0.021
ALS rare variant carrier	30 (20.7)	12 (15.0)	18 (27.7)	2.16 [0.89–5.42]	0.067
Braak NFT stage					
Braak 0	23 (16.0)	17 (21.5)	6 (9.2)	–	0.20
Braak I	39 (27.1)	22 (27.8)	17 (26.2)		
Braak II	60 (41.7)	29 (36.7)	31 (47.7)		
Braak III	18 (12.5)	8 (10.1)	10 (15.4)		
Braak IV	2 (1.4)	2 (2.5)	0 (0)		
Braak V	2 (1.4)	1 (1.3)	1 (1.5)		
Braak VI	0 (0)	0 (0)	0 (0)		
Thal Aβ phase					
Phase 0	73 (50.7)	48 (60.8)	25 (38.5)	–	0.094
Phase 1	32 (22.2)	13 (16.5)	19 (29.2)		
Phase 2	12 (8.3)	6 (7.6)	6 (9.2)		
Phase 3	23 (16.0)	11 (13.9)	12 (18.5)		
Phase 4	1 (0.7)	0 (0)	1 (1.5)		
Phase 5	3 (2.1)	1 (1.3)	2 (3.1)		

*y* years, m months, *NFT* neurofibrillary tangle

The most common APOE genotype was ε3/ε3, observed in 102 cases (70.3%). Sixteen cases (11.0%) were ε2 carriers, and 29 cases (20.0%) were ε4 carriers. One hundred fifteen cases (79.3%) harbored no RV in known ALS-associated genes, whereas 30 cases (20.7%) carried one or more RV. The genes in which RV were identified and the number of rare variants detected in each gene are listed in Supplementary Table 2. Most cases included in the present study were also part of a separate manuscript currently under review elsewhere, in which detailed information on the RV is provided. It should be noted that AD Braak staging could not be assessed in one case.

### Association of APOE status with TDP-43 pathological subtypes and clinical characteristics in ALS

We first examined whether *APOE* allele status was associated with ALS TDP-43 pathology subtype by comparing the proportions of ALS types 1 and 2 between *APOE* ε2/ε4 carriers and non-carriers. There were 29 *APOE* ε4 carriers, of whom 10 (34.5%) were classified as type 1 and 19 (65.5%) as type 2. In contrast, among 116 *APOE* ε4 non-carriers, 70 (60.3%) were type 1 and 46 (39.7%) were type 2. There were no significant differences in age at onset, survival time, prevalence of dementia, or frequency of bulbar onset according to APOE ε4 or APOE ε2 carrier status (Supplementary Tables 3 and 4). However, *APOE* ε4 carriers had a significantly higher proportion of type 2 pathology than non-carriers (Fisher’s exact test, p = 0.021; odds ratio 2.87, 95% CI 1.14–7.58) (Table 1). In contrast, no association was observed between APOE ε2 carrier status and ALS subtype proportions. Based on these findings, we next used SEM and random forest to further investigate the relationship between APOE4 and ALS pathological subtype in a more comprehensive manner.

### Initial SEM and random forest model

To further investigate the relationship between APOE ε4 and ALS pathological subtype while accounting for potential confounders, we performed SEM. Temporal clinical parameters were defined in two ways. Model (a) included age at death and entire course, including the period after initiation of mechanical ventilation, to account for the potential influence of aging at autopsy and continued pathological progression after ventilation. Model (b) incorporated age at onset and survival time, defined as the time from symptom onset to initiation of mechanical ventilation or death without ventilation, focusing on clinically relevant parameters.

The initial Bayesian SEM (a) was fitted, yielding a PPP of 0.25, indicating an adequate model fit. The results are shown in Fig. 2a and Table 2. SEM revealed that *APOE* ε4 and age at death significantly contributed to both Thal Aβ phase and ALS TDP-43 pathology subtypes. While Thal Aβ phase and age at death drove the progression of Braak NFT stage, the impact of *APOE* ε4 on the pathological distribution of TDP-43 inclusions in ALS was not supported as an indirect pathway mediated by these AD-related markers.

**Table 2:** Results from the initial bayesian structural equation model (a)

Association	Estimate	posterior interval	standardized factor loadings
Thal Aβ phase			
APOE4	0.99*	0.55 – 1.42	0.38
RV	0.29	− 0.18 – 0.74	0.11
Age at death	0.03*	0.01 – 0.05	0.25
Entire course	− 0.00	− 0.00 – 0.00	− 0.09
Braak NFT stage			
APOE4	− 0.30	− 0.77 – 0.14	− 0.11
Thal Aβ phase	0.35*	0.13 – 0.57	0.33
RV	0.35	− 0.07 – 0.81	0.12
Age at death	0.04*	0.02 – 0.06	0.34
Antire course	− 0.00	− 0.00 – 0.00	− 0.04
TDP-43 type2			
APOE4	0.70*	0.13 – 1.33	0.27
Thal Aβ phase	0.02	− 0.27 – 0.32	0.02
Braak NFT stage	− 0.05	− 0.30 – 0.21	− 0.06
RV	0.44	− 0.11 – 1.00	0.16
Age at death	0.03*	0.01 – 0.06	0.29
Entire course	− 0.00	− 0.01 – 0.00	− 0.16

*RV* rare variant, *NFT* neurofibrillary tangles

^*^ estimates whose 95% confidence interval does not cross 0

Thal Aβ phase was influenced by APOE4 (estimate [posterior interval], standardized factor loading: 0.99 [0.55–1.42], 0.38) and age at death (0.03 [0.01–0.05], 0.25), indicating that a higher number of *APOE* ε4 alleles and older age at death were associated with more predominant amyloid-β pathology. Braak NFT stage was influenced by Thal Aβ phase (0.35 [0.13–0.57], 0.33) and age at death (0.04 [0.02–0.06], 0.34), demonstrating that more advanced amyloid-β pathology and older age at death were associated with more predominant tau pathology. ALS TDP-43 pathology subtype was influenced by APOE4 (0.70 [0.13–1.33], 0.27) and age at death (0.03 [0.01–0.06], 0.29), suggesting that higher APOE4 values and older age at death were associated with a higher likelihood of type 2 pathology. In contrast, the paths from Thal Aβ phase and Braak NFT stage to ALS subtype had 95% credible intervals that included zero, and indirect pathways mediated through amyloid-β or tau pathology were not statistically supported in this dataset. RV in ALS-associated genes showed no clear effects on Thal Aβ phase, Braak NFT stage, or ALS TDP-43 pathology subtype. Estimation using ULSMV yielded consistent directions of effect, confirming the robustness of the findings (Supplementary Table 5).

In addition, we applied a random forest model as a non-pathway-based, unbiased approach and performed analyses using both definitions of temporal clinical parameters (a) and (b). For temporal clinical parameters (a), the mean AUC obtained by fivefold cross-validation was 0.69. The variables with the highest importance for predicting TDP-43 type were entire course, APOE ε4, RV, age at death, Thal Aβ phase, and Braak NFT stage, in descending order (Supplementary Table 6). However, entire course was not significantly associated with TDP-43 type 2 in the SEM analysis. Partial dependence plot analysis showed no simple correlation between entire course and the predicted probability of TDP-43 type 2 (Supplementary Fig. 1a). In contrast, a higher number of APOE ε4 alleles was associated with a higher predicted probability of TDP-43 type 2 (Supplementary Fig. 1a).

**Fig. 3:**
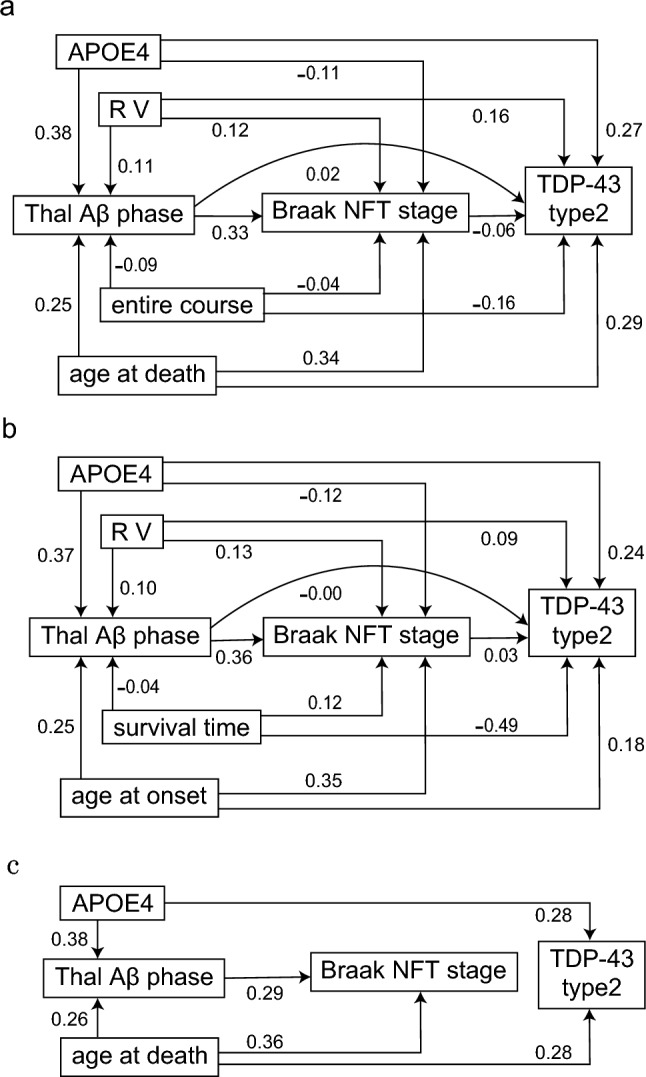
(a)Initial Bayesian SEM model (**a**). All possible pathways were examined. (b)Initial Bayesian SEM model (**b**). All possible pathways were examined. (c)Final Bayesian SEM model (**a**). The model was refined to include only pathways that showed statistically significant effects in the initial model (**a**), defined as those whose 95% credible intervals did not include zero. The numbers shown next to the arrows indicate standardized factor loadings. Aβ amyloid-β, NFT neurofibrillary tangle, RV rare variant

The initial Bayesian SEM (b) also showed adequate model fit (PPP = 0.47). The results are shown in Fig. 3b and Table 3. Consistent with SEM (a), APOE ε4 significantly contributed to both Thal Aβ phase and ALS TDP-43 pathology subtype, while no indirect effect via AD-related markers on TDP-43 subtype was supported. Thal Aβ phase was influenced by APOE4 (0.97 [0.54–1.40], 0.37) and age at onset (0.02 [0.01–0.04], 0.25), indicating that a higher number of *APOE* ε4 alleles and older age at onset were associated with more predominant amyloid-β pathology. Braak NFT stage was influenced by Thal Aβ phase (0.38 [0.17–0.61], 0.36) and age at onset (0.04 [0.02–0.05], 0.35), demonstrating that more advanced amyloid-β pathology and older age at death were associated with more predominant tau pathology. ALS TDP-43 pathology subtype was influenced by APOE4 (0.73 [0.16–1.37], 0.24) and survival time (−0.02 [−0.03– −0.01], −0.49), indicating that a greater number of APOE ε4 alleles and poor prognosis were associated with a greater likelihood of type 2 pathology, characterized by spread into non-motor regions.

**Table 3:** Results from the initial bayesian structural equation model (b)

Association	Estimate	posterior interval	standardized factor loadings
Thal Aβ phase			
APOE4	0.97*	0.54 – 1.40	0.37
RV	0.28	− 0.18 – 0.72	0.10
Age of onset	0.02*	0.01 – 0.04	0.25
Survival time	− 0.00	− 0.01 – 0.01	− 0.04
Braak NFT stage			
APOE4	− 0.33	− 0.81 – 0.14	− 0.12
Thal Aβ phase	0.38*	0.17 – 0.61	0.36
RV	0.36	− 0.10 – 0.81	0.13
Age of onset	0.04*	0.02 – 0.05	0.35
Survival time	0.00	− 0.00 – 0.01	0.12
TDP-43 type2			
APOE4	0.73*	0.16 – 1.37	0.24
Thal Aβ phase	− 0.00	− 0.30 – 0.30	− 0.00
Braak NFT stage	0.04	− 0.22 – 0.29	0.03
RV	0.28	− 0.27 – 0.83	0.09
Age of onset	0.02	− 0.00 – 0.04	0.18
Survival time	− 0.02*	− 0.03 – − 0.01	− 0.49

*RV* rare variant, *NFT* neurofibrillary tangles

^*^ estimates whose 95% confidence interval does not cross 0

Furthermore, using temporal clinical parameters (b), the mean AUC obtained by five-fold cross-validation was 0.76. The variables with the highest importance for predicting TDP-43 type were survival time, age at onset, number of APOE ε4 alleles, Braak NFT stage, Thal Aβ phase, and RV, in descending order (Supplementary Table 7). Partial dependence plot analysis showed that a shorter survival time and a higher number of APOE ε4 alleles were associated with a higher predicted probability of TDP-43 type 2, consistent with the results of SEM analysis (Supplementary Fig. 1b).

Importantly, the association between APOE ε4 and ALS TDP-43 subtype persisted after amyloid-β and tau pathology indicators had been incorporated in the same SEM framework, supporting a direct effect within the specified model rather than an indirect pathway mediated by AD-related pathologies (Fig. 3, Tables 2,3,4).

**Table 4:** Results from the final bayesian structural equation model

Association	Estimate	posterior interval	standardized factor loadings
Thal Aβ phase			
APOE4	0.99*	0.56 – 1.41	0.38
Age at death	0.03*	0.01 – 0.05	0.26
Braak NFT stage			
Thal Aβ phase	0.30*	0.12 – 0.50	0.29
Age at death	0.04*	0.02 – 0.06	0.36
TDP-43 type2			
APOE4	0.72*	0.18 – 1.28	0.28
Age at death	0.03*	0.01 – 0.05	0.28

*RV* rare variant, *NFT* neurofibrillary tangles

^*^ estimates whose 95% confidence interval does not cross 0

### Final SEM (a) model

Bayesian SEM (a) was re-estimated using only the paths whose 95% credible intervals did not include zero in the initial model, yielding the final model (Fig. 3c and Table 4). The PPP was 0.45, indicating an adequate model fit. In the final model, Thal Aβ phase was influenced by APOE4 (0.99 [0.56–1.41], 0.38) and age at death (0.03 [0.01–0.05], 0.26). Braak NFT stage was influenced by Thal Aβ phase (0.30 [0.12–0.50], 0.29) and age at death (0.04 [0.02–0.06], 0.36). ALS TDP-43 pathology subtype was influenced by APOE4 (0.72 [0.18–1.28], 0.28) and age at death (0.03 [0.01–0.05], 0.28).

### SEM and random forest analysis of determinants of age at death

A separate SEM was constructed with age at death as the endogenous variable to explore whether neuropathological features influence lifespan in ALS. The model demonstrated that TDP-43 type 2 pathology (2.81 [1.03—4.70], 0.30) and Braak NFT stage (3.46 [1.83—5.06], 0.39) were significantly associated with older age at death, whereas APOE ε4, Thal Aβ phase, and RV did not show direct effects (Supplementary Table 8, Supplementary Fig. 2). In the random forest analysis, the predictive performance for age at death showed a mean RMSE of 9.00, MAE of 7.20, and R^2^ of 0.14. The variables with the highest importance for predicting age at death, as assessed by %IncMSE, were Braak NFT stage, TDP-43 type 2, Thal Aβ phase, APOE ε4, and RV, in descending order (Supplementary Table 9). Partial dependence plot analysis indicated that higher Braak NFT stage and the presence of TDP-43 type 2 pathology were associated with a higher predicted age at death (Supplementary Fig. 1c).

## Discussion

Using autopsy-confirmed cases of sporadic ALS, we have shown that *APOE* ε4 is associated with a widespread (type 2) distribution pattern of TDP-43 pathology. In the present study, temporal clinical parameters were defined using two alternative approaches: (a) a model that accounts for continued neurodegeneration after the initiation of mechanical ventilation (entire course), and (b) a model that more closely reflects the clinical disease course (survival time). These two parameter settings were evaluated using both SEM and random forest analysis, and consistent results were obtained across both models, demonstrating an association between APOE ε4 and the type 2, widespread distribution of TDP-43 pathology. These findings support the notion that APOE ε4 may contribute to the distribution pattern of TDP-43 pathology in ALS.

Comparison of the results obtained from random forest and SEM analyses revealed broadly similar trends, although some discrepancies were observed. In model (a), random forest analysis identified entire course as a variable with high importance, whereas SEM did not demonstrate a significant association between entire course and the widespread (type 2) distribution of TDP-43 pathology. To further investigate this discrepancy, partial dependence plots were generated, which showed no simple linear relationship between entire course and the predicted probability of TDP-43 type 2. Thus, this may reflect the ability of random forest to capture nonlinear relationships, whereas SEM did not detect a statistically significant association. Aside from this discrepancy, the findings from SEM and random forest were largely consistent, supporting the robustness of the main results of this study.

Regarding clinical correlations, in this cohort, TDP-43 type 2 cases tended to show older age at onset, a more rapidly progressive disease course, and a higher frequency of dementia. Furthermore, in both SEM and random forest analyses of model (b), shorter survival time was identified as a factor associated with the widespread distribution of TDP-43 pathology. Taken together, these findings suggest that widespread TDP-43 pathology in ALS is associated with a clinical phenotype characterized by older onset, rapid progression, and cognitive impairment.

In contrast, when APOE ε4 carriers and non-carriers were compared, no statistically significant differences in clinical characteristics were observed in this cohort. Previous studies examining the association between APOE ε4 and prognosis in ALS have reported inconsistent findings, with some studies suggesting an association with poorer prognosis and others failing to demonstrate such a relationship [19–21]. In addition, earlier studies have suggested that APOE ε4 may be associated with bulbar-onset ALS [22]; however, we did not observe a clear association between APOE genotype and bulbar onset in the present study (Supplementary Table 3). This may in part be attributable to the limited sample size in the present cohort, and further validation in larger cohorts is warranted to clarify the relationship between APOE ε4 and the clinical course of ALS.

Previous studies have established that APOE ε4 is a major genetic determinant of amyloid-β (Aβ) deposition [23]. Proposed mechanisms include reduced clearance of Aβ from the brain [24] and promotion of Aβ aggregation and seeding [25]. APOE ε4 has also been reported to be associated with NFTs, although this effect is weaker than that on plaques and is considered to be largely secondary to Aβ. Indeed, SEM analyses of AD pathology have shown that APOE ε4 promotes plaque formation, which in turn promotes NFTs, whereas no direct effect of APOE ε4 on NFTs was observed [9]. In contrast, studies using P301S tau transgenic mice have suggested that APOE ε4 may promote tau accumulation independently of Aβ [26]). Taken together, APOE ε4 appears to exert a stronger influence on Aβ than on tau, with much of its effect on tau pathology mediated indirectly through Aβ. In the present SEM analysis, APOE ε4 was also associated with increased Aβ, with downstream effects on tau pathology, suggesting that these relationships are preserved in the brains of patients with ALS. In contrast, the effect of APOE ε4 on the widespread distribution of TDP-43 pathology was not clearly mediated by coexisting pathologies such as Aβ or tau but was instead suggested to be a direct effect. Instead, *APOE* ε4 retained an effect on ALS TDP-43 subtype even when AD-related pathological indicators and clinical indicators were modeled concurrently.

The direct involvement of *APOE* ε4 in the pathological distribution of TDP-43 inclusions in ALS represents a crucial step towards the molecular stratification of the disease. The lack of an association between AD-related pathological markers and ALS-associated TDP-43 inclusion distribution patterns suggests a distinct mechanistic divergence between AD and ALS.

A potential explanation for this direct effect lies in the pleiotropic immunomodulatory functions of APOE [10]. Recent large-scale proteomic studies have identified a conserved *APOE* ε4-associated protein signature enriched for pro-inflammatory pathways, which appears largely independent of classical neuropathological markers. This biological vulnerability conferred by *APOE* ε4 may promote the dissemination of TDP-43 pathology in the ALS brain through altered neuroinflammatory environments rather than through the facilitation of amyloid or tau seeding [10].

Our findings contrast with an AD-based study where APOE ε4 was found to exert both direct and amyloid-mediated indirect effects on TDP-43 [9]. This discrepancy likely reflects not only our focus on the anatomical distribution pattern (type 1 vs type 2) in ALS rather than the simple presence or absence of TDP-43 inclusions, but also fundamental differences in the underlying disease context, as our present study specifically addresses ALS rather than AD. Collectively, this suggests that the mechanisms governing the systematic spread of TDP-43 inclusions in ALS are at least partially distinct from those in AD.

The extent of TDP-43 pathology can generally only be determined by post-mortem neuropathological evaluation. However, if the presence of APOE ε4—which can be assessed during life—suggests a higher likelihood of widespread pathology (TDP-43 type 2), this information may facilitate earlier clinical decision-making. For example, early cognitive assessment, timely discussions regarding treatment decisions such as ventilatory support, advance care planning, and earlier introduction of communication support devices may facilitate care that better reflects the patient’s preferences.

Several study limitations warrant consideration. First, the number of APOE ε4 carriers was modest, and the effect estimates will need to be validated in independent cohorts. Second, the pathological outcome was based on a binary pattern classification and did not evaluate quantitatively differences in the extent of TDP-43 pathology within each group. Third, SEM evaluates the consistency of hypothesized relationships in observational data and does not establish causality; unmeasured confounding and alternative model specifications remain possible. Finally, methods for detecting TDP-43 pathology have advanced rapidly in recent years, with a variety of approaches being reported, including aptamer-based techniques, cryptic exon-targeted in situ hybridization probes, PCR-based detection of cryptic exons, and cryptic peptide-specific antibodies. A limitation of the present study is that the presence of TDP-43 pathology was assessed solely by phosphorylated TDP-43 immunohistochemistry (pTDP-43 IHC). However, the primary aim of this study was not to establish the presence of TDP-43 pathology per se, but rather to identify factors that determine its distribution pattern. Given that pTDP-43 IHC remains the current standard method for evaluation of TDP-43 pathology, this approach was considered appropriate for the present purposes. Future studies incorporating these emerging detection techniques, including analyses of the relationship between the APOE ε4 allele and TDP-43 distribution patterns using more sensitive methodologies, will be important for further refining our understanding of TDP-43 pathology.

While the present study focused on TDP-43 pathology within the central nervous system, recent studies have begun to explore the extent of more widespread involvement, including peripheral tissues [27], with emerging evidence also suggesting involvement of skin and gastrointestinal tissues [28], representing an important area for future investigation. APOE is known to influence peripheral tissues through its roles in lipid metabolism and inflammatory responses [29], raising the possibility that APOE genotype may also affect the distribution of TDP-43 pathology outside the central nervous system. However, in the present study, tissue samples from peripheral sites such as the skin and gastrointestinal tract were limited, precluding additional analyses. Notably, skin tissue can be obtained relatively easily during life, and future studies in larger cohorts examining the relationship between APOE genotype and peripheral TDP-43 pathology are warranted.

In conclusion, *APOE* ε4 status is associated with the widespread TDP-43 pathological subtype evident in sporadic ALS. Integration of *APOE* genotyping into clinical and pathological assessments may improve prognostic stratification and facilitate the development of targeted therapeutic strategies tailored to specific molecular subtypes of ALS.

## Supplementary Information

Below is the link to the electronic supplementary material.

Supplementary Fig. 1. Partial dependence plots showing the marginal effect of each variable on the predicted outcome in the random forest model. The effect of each variable is estimated while averaging over the joint distribution of the other variables in the dataset. (a) Random forest model (a) for the classification of TDP-43 pathological type. The y-axis indicates the predicted type 2 positive probability, and the x-axis represents the variable of interest. (b) Random forest model (b) for the classification of TDP-43 pathological type. The y-axis indicates the predicted type 2 positive probability, and the x-axis represents the variable of interest. (c) Random forest model for the analysis of determinants of age at death. The y-axis indicates the predicted age at death, and the x-axis represents the variable of interest. Aβ: amyloid-β, NFT: neurofibrillary tangle, RV: rare variant. Supplementary file1 (PDF 1229 KB)

Supplementary Fig. 2. Bayesian SEM model for the analysis of determinants of age at death. All possible pathways were examined. Aβ: amyloid-β, NFT: neurofibrillary tangle, RV: rare variant. Supplementary file2 (PDF 444 KB)

Supplementary file3 (XLSX 21 KB)

## Data Availability

The datasets used and/or analyzed during the present study are available from the corresponding author on reasonable request.
