# Supplementary figures and images for: APOE ε4 influences the widespread TDP-43 pathological subtype in sporadic amyotrophic lateral sclerosis
Source: Acta Neuropathol. 2026 May 15;151(1):57. doi: 10.1007/s00401-026-03029-y (PMC13179199; doi:10.1007/s00401-026-03029-y)

a

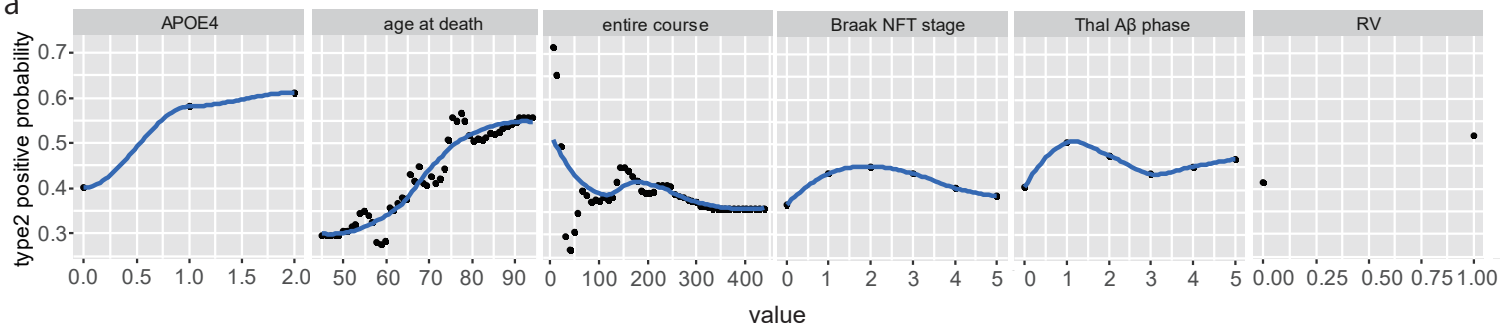

b

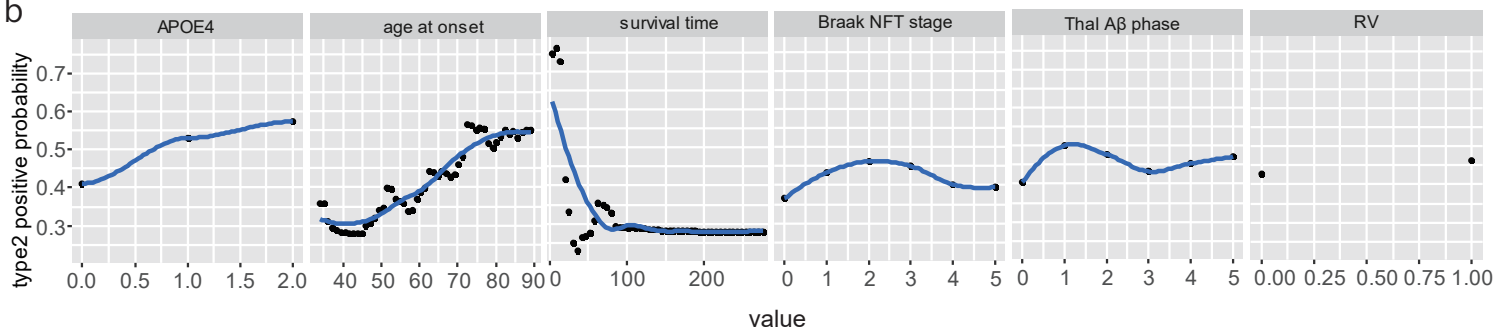

c

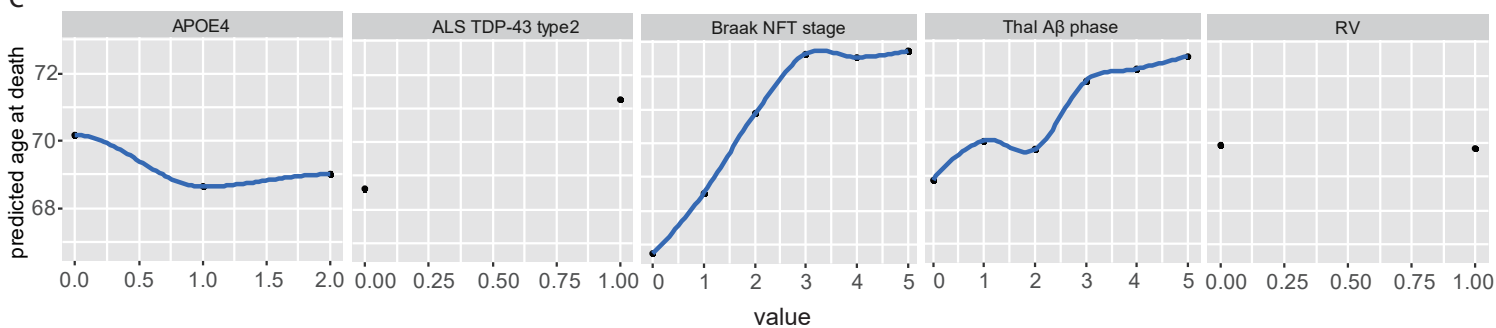

Supplement: Supplementary file 1 — Supplementary Fig. 1. Partial dependence plots showing the marginal effect of each variable on the predicted outcome in the random forest model. The effect of each variable is estimated while averaging over the joint distribution of the other variables in the dataset. (a) Random forest model (a) for the classification of TDP-43 pathological type. The y-axis indicates the predicted type 2 positive probability, and the x-axis represents the variable of interest. (b) Random forest model (b) for the classification of TDP-43 pathological type. The y-axis indicates the predicted type 2 positive probability, and the x-axis represents the variable of interest. (c) Random forest model for the analysis of determinants of age at death. The y-axis indicates the predicted age at death, and the x-axis represents the variable of interest. Aβ: amyloid-β, NFT: neurofibrillary tangle, RV: rare variant. Supplementary file1 (PDF 1229 KB) [file 401_2026_3029_MOESM1_ESM.pdf]

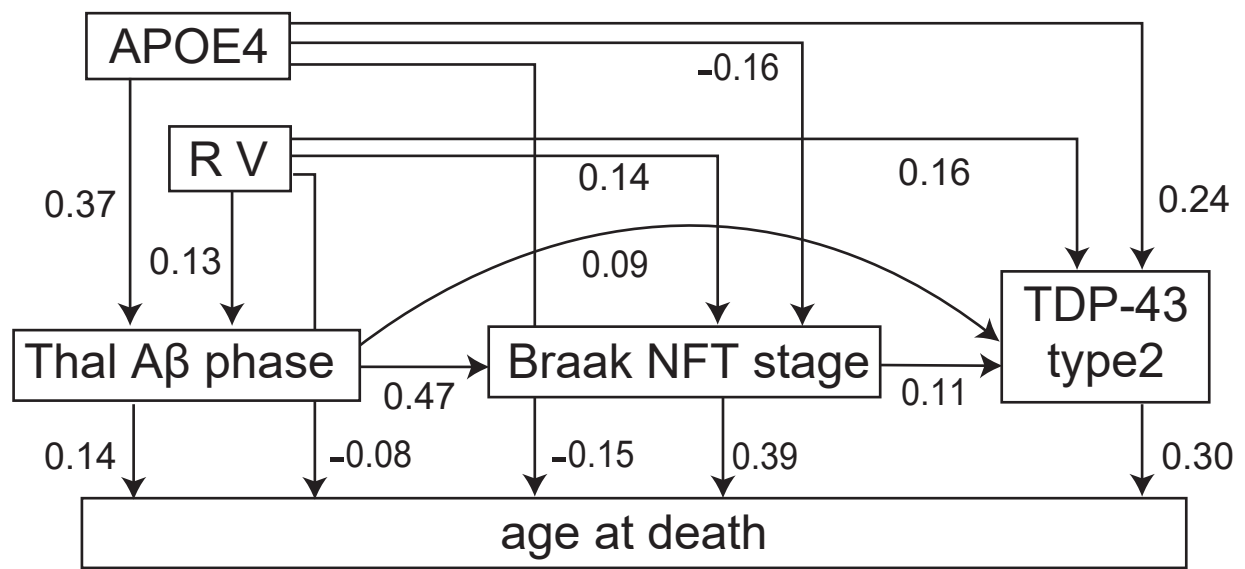

Supplement: Supplementary file 2 — Supplementary Fig. 2. Bayesian SEM model for the analysis of determinants of age at death. All possible pathways were examined. Aβ: amyloid-β, NFT: neurofibrillary tangle, RV: rare variant. Supplementary file2 (PDF 444 KB) [file 401_2026_3029_MOESM2_ESM.pdf]
